# An Extrinsic Fabry Perot Fiber Optic Current Transformer Based on PZT Coupling

**DOI:** 10.3390/mi17070806

**Published:** 2026-07-01

**Authors:** Shiguang Bai, Zhongyuan Li, Yanju Li, Qichao Chen

**Affiliations:** 1 Datang Northeast Electric Power Test & Research Institute Co., Ltd., Changchun 130102, China; 17390019535@163.com; 2 Electric Power Research Institute, State Grid Heilongjiang Electric Power Co., Ltd., Harbin 150030, China; li8zhongyuan@126.com; 3 State Key Laboratory of High-Efficiency Special Cable Technology, Harbin University of Science and Technology, Harbin 150080, China

**Keywords:** fiber-optic current transformer, extrinsic Fabry–Perot interferometer, lead zirconate titanate piezoelectric ceramic, intensity demodulation, low-current detection, static operating point control

## Abstract

To address the structural complexity, limited detection sensitivity, and environmental susceptibility of the stable operating point in conventional fiber-optic current transformers for low-current detection, this study proposes a fiber-optic current transformer based on the coupling of an extrinsic Fabry–Perot interferometer (EFPI) and a lead zirconate titanate piezoelectric ceramic (PZT). In the proposed sensor, a toroidal magnetic core and an induction winding are used as the current pickup unit to convert the measured alternating current into an induced voltage. This induced voltage directly drives the PZT to generate axial displacement, causing periodic variations in the length of the air Fabry–Perot cavity formed between the fiber end face and the coated quartz diaphragm. As a result, the current signal is converted into an optical interference intensity signal. To prevent the static operating point from deviating from the optimal linear region during EFPI intensity demodulation, a DC-component-feedback-based operating point control method is proposed. By adjusting the driving voltage of the fiber Fabry–Perot tunable filter, the center wavelength of the incident narrowband demodulation light can track the optimal operating point of the interference spectrum, thereby improving the stability of the intensity demodulation process. Experimental results show that the fabricated sensor can generate a stable reflected interference spectrum and exhibits a relatively flat frequency response within the range of 0–7 kHz, indicating its potential for power-frequency current detection under the present laboratory conditions. When the measured current is 0.13 mA, the sensor can still produce a distinguishable sinusoidal output signal. When the measured current increases to 75 mA, obvious nonlinear distortion appears in the output signal, indicating that the sensor is approaching the boundary of its linear detection range. Within the linear operating region, the output peak-to-peak value shows good linearity with the measured current. The results indicate that the proposed EFPI-PZT fiber-optic current transformer has the advantages of a relatively simple structure, clear low-current response, and adjustable structural parameters, providing a reference for the miniaturized design and further development of new fiber-optic current sensors.

## 1. Introduction

With the continuous expansion of power systems and the increasing demand for long-distance power transmission, high-penetration renewable energy sources and power electronic devices have been widely integrated into power grids. As a result, power-system operation has become more stochastic, fluctuating, and complex [1]. In this context, higher requirements have been imposed on current detection technologies in terms of measurement accuracy, real-time response, insulation performance, and intelligent interconnection capability. Although conventional electromagnetic current transformers have been widely used, their detection performance is susceptible to core saturation, remanence, insulation structure, and complex electromagnetic environments. In addition, detection methods that rely on manual inspection and secondary electrical transmission can no longer fully meet the requirements of modern smart grids for current-state awareness. With the rapid development of fiber-optic sensing and demodulation technologies, optical current transformers have attracted extensive attention from researchers worldwide because of their high measurement accuracy, strong immunity to electromagnetic interference, excellent insulation performance, long transmission distance, and suitability for cascading, interconnection, and rapid networking [2,3].

Conventional fiber-optic current transformers can generally be classified into all-fiber, optoelectronic hybrid, and electromagnetic-sensitive-material-coupled types [4]. Among them, all-fiber current sensors are mostly developed based on the Faraday magneto-optic effect. They offer advantages such as simple structure, long service life, and good insulation performance of the sensing unit. However, their detection accuracy and stability are easily affected by linear birefringence, polarization-state drift, ambient temperature variation, and external vibration, making it difficult to fully ensure long-term stability in engineering applications [5,6]. Yu et al. proposed an all-fiber current sensor based on a phase-shift fiber-loop ringdown structure, in which the current detection sensitivity was improved by allowing the optical signal to propagate multiple times in the sensing fiber. An optimal current sensitivity of 0.8158°/A and a minimum detectable current of 100 mA were experimentally obtained [7]. Zhang et al. proposed a resonant fiber-optic current sensor based on a broadband light source and a single-mode fiber ring resonator. Current detection was achieved by using the resonant frequency difference between two circularly polarized light beams, yielding a sensitivity of 41.53 Hz/A and a relative error below 0.2% over the range of 0–400 A [8]. Although these studies improved the sensitivity and detection accuracy of all-fiber current sensors, the demodulation process remains closely related to the polarization state, optical-path stability, and environmental disturbances. Therefore, the low-current detection capability still requires further improvement.

Optoelectronic hybrid current transformers usually employ a conventional current transformer, Rogowski coil, or power pickup coil as the current sensing unit, while optical fiber or fiber Bragg grating is used as the signal transmission and demodulation carrier. Such structures can, to some extent, combine the advantages of conventional electromagnetic induction units and fiber-optic transmission. However, electromagnetic energy pickup, signal conversion, electronic conditioning, or optoelectronic conversion is still involved in the sensing chain, leading to a complex system structure and limited long-term operational reliability [9]. Mir et al. proposed a passive autoranging circuit for a hybrid FBG/PZT photonic current transducer. The structure consists of a current transformer, a piezoelectric transducer, and an FBG, and range extension is achieved using a series load resistor and MOSFET switching. Fast responses at thresholds of approximately 2 A and 20 A were experimentally demonstrated [10]. Fusiek et al. further tested the passive autoranging method for this type of photonic current transducer. The results showed that the structure could use a CT, PZT, and FBG for multipoint current measurement in power grids, with range switching achieved at thresholds of 130% rated current and 22 times rated current, and with a response time below 4 ms [11]. Nevertheless, this type of system still requires the coordinated operation of a current transformer, load resistor, protection circuit, PZT transducer, and FBG demodulation unit. The system structure and signal chain are relatively complex, and the dynamic range of the FBG/PZT structure is also limited by the voltage withstand capability of the piezoelectric transducer and the noise floor of the demodulator [12].

Current transformers based on the magnetostrictive effect usually combine fiber Bragg gratings (FBGs) with giant magnetostrictive materials (GMMs). The magnetic field generated by the measured current drives the sensitive material to produce magnetostriction or refractive-index variation, and the current magnitude is then indirectly reflected by the center-wavelength shift or optical-intensity variation in the FBG. This results in a complicated sensing-unit structure, and the required bias magnetic-field strength limits the current detection range, making practical engineering application less convenient [13]. Jiao et al. improved the performance of a GMM-FBG current sensor by optimizing the grating-spectrum parameters. Experimental results showed that the optimized sensor achieved a linearity of 0.9942, a sensitivity of 249.75 mV/A, and good stability over the temperature range of 0–60 °C [14]. Zhuang et al. proposed a fiber-optic current sensing system based on optical-carrier microwave interferometry and the virtual Vernier effect, in which an FBG bonded to Terfenol-D giant magnetostrictive material was used as the sensing unit, enabling tunable sensitivity and high current resolution [15].

In addition, some researchers have used the inverse piezoelectric effect of stacked piezoelectric ceramics, in which the displacement response is proportional to the applied voltage, and coupled this structure with an FBG for current measurement. However, because the strain sensitivity of FBGs is relatively low and the electromagnetic energy pickup unit cannot directly provide a high voltage for the PZT, power electronic devices are required to rectify the AC signal before driving the PZT. The PZT then drives the coupled FBG to perform current detection. Moreover, the detection system requires a distributed fiber-optic sensing network analyzer, which makes the current-transformer sensing system highly complex and unfavorable for engineering application [16,17]. Apart from the above detection methods, some studies have fixed a permanent magnet to the outer end of a constant-strain cantilever beam and coupled an FBG to the beam. Current detection is realized by using the interaction between the electromagnetic force generated by an AC magnetic field and the permanent magnet to drive cantilever vibration. This sensing unit requires an additional energized solenoid structure to guide the AC electrical signal, resulting in a large sensor volume, complex structure, and poor stability [18]. These studies indicate that coupling FBGs with electromagnetic sensitive materials can effectively realize current detection. However, such structures are usually affected by magnetostrictive hysteresis, temperature drift, the requirement for a bias magnetic field, and the limited microstrain response sensitivity of FBGs, leading to remaining limitations in low-current detection and engineering implementation.

In summary, existing fiber-optic current transformers have significant advantages in electromagnetic-interference immunity and electrical insulation. However, all-fiber sensors are susceptible to polarization-state variation and environmental disturbances, optoelectronic hybrid sensors suffer from complex signal chains, and electromagnetic-sensitive-material-coupled sensors mostly rely on the wavelength response of FBGs to microstrain. The intrinsic strain-response sensitivity of FBGs limits their capability for detecting low-current signals. Therefore, it is necessary to develop a new fiber-optic current transformer with a compact structure, high sensitivity, no requirement for an external bias magnetic field, and suitability for low-current detection.

To further clarify the performance position of the proposed sensor, a concise comparison with representative optical current sensors is provided here. Yu et al. reported an all-fiber current sensor based on a phase-shift fiber-loop ringdown structure, achieving a current sensitivity of 0.8158°/A and a minimum detectable current of 100 mA. Zhang et al. proposed a resonant fiber-optic current sensor based on a single-mode fiber ring resonator, obtaining a sensitivity of 41.53 Hz/A and a relative error below 0.2% over the range of 0–400 A. These all-fiber current sensors show good insulation performance and measurement accuracy, but their demodulation processes are still affected by polarization-state stability, optical-path stability, and environmental disturbances. For hybrid FBG/PZT photonic current transducers, Mir et al. demonstrated passive autoranging responses at approximately 2 A and 20 A, and Fusiek et al. further reported range switching at 130% rated current and 22 times rated current with a response time below 4 ms. These methods are useful for range extension, but they usually require the coordinated operation of a current transformer, load resistor, protection circuit, PZT transducer, and FBG demodulation unit. In addition, Jiao et al. reported a GMM-FBG current sensor with a sensitivity of 249.75 mV/A, a linearity of 0.9942, and stable performance over 0–60 °C; however, such structures are still affected by the characteristics of magnetostrictive materials, temperature drift, and bias-field-related limitations.

Compared with the above studies, the purpose of this work is not to provide a complete replacement for all existing optical current sensors, but to propose and verify a relatively simple EFPI-PZT current-sensing route for low-current response. In the proposed structure, the measured current is first converted into an induced voltage by a toroidal magnetic core and an induction winding, and the voltage directly drives the PZT to modulate the EFPI cavity length. Under the present experimental conditions, the fabricated sensor produced a distinguishable sinusoidal output at 0.13 mA and exhibited a relatively flat frequency response within 0–7 kHz. When the measured current increased to 75 mA, waveform distortion appeared, indicating that the sensor was approaching the upper boundary of its linear operating region. Therefore, the present results demonstrate the feasibility and low-current response potential of the EFPI-PZT sensing method, while the linear dynamic range, long-term stability, temperature adaptability, and engineering packaging still require further optimization.

An extrinsic Fabry–Perot interferometer (EFPI) fiber-optic sensor can convert minute cavity-length variations into interference-intensity changes. It features a simple structure, high sensitivity, strong immunity to electromagnetic interference, and ease of integration. Meanwhile, lead zirconate titanate piezoelectric ceramic (PZT) exhibits favorable inverse piezoelectric deformation under an AC driving voltage and can effectively convert electrical signals into mechanical displacement. Therefore, in this work, the EFPI sensing structure is coupled with a PZT actuator. A toroidal magnetic core and an induction winding are used to convert the measured alternating current into an induced voltage, which then drives the PZT to produce periodic deformation. This deformation causes periodic variations in the Fabry–Perot cavity length between the fiber end face and the coated quartz diaphragm, thereby converting the measured current signal into an optical interference signal. This study first analyzes the interference sensing mechanism of the EFPI fiber-optic sensor. Then, an EFPI fiber-optic current-transformer sensing structure driven by PZT is designed, and a sensing detection and demodulation system is established. The basic characteristics, current detection performance, and operating point control method of the sensor are investigated, aiming to explore a compact EFPI-PZT fiber-optic current-sensing route with clear low-current response and adjustable structural parameters.

## 2. Principles and Design of the Fiber-Optic Current Transformer

### 2.1. Interference Sensing Mechanism of the Extrinsic Fabry–Perot Interferometer

**Figure 1:**
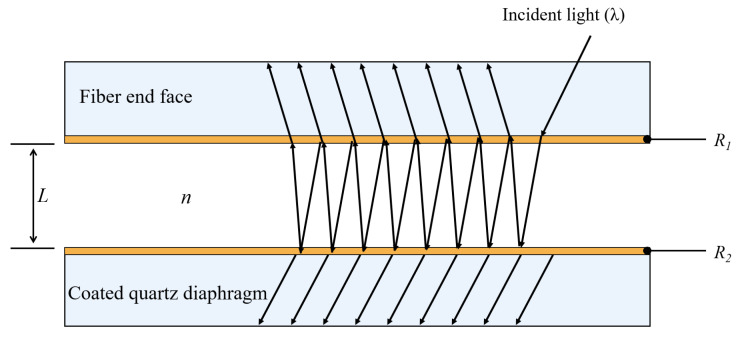
Fabry–Perot cavity interference principle.

An extrinsic Fabry–Perot interferometer (EFPI) fiber-optic sensor is typically composed of a fiber end face, an air cavity, and a coated quartz diaphragm. The Fabry–Perot cavity is formed between the fiber end face and the external reflective surface. After the optical signal emitted from the light source is transmitted to the sensing end through the optical fiber, part of the light is reflected at the fiber end face, while the remaining part enters the air cavity and is reflected by the surface of the coated quartz diaphragm. The light beams reflected from the two reflective surfaces are recoupled into the fiber and form a cavity-length-dependent interference signal. Since variations in the Fabry–Perot cavity length cause changes in the phase difference in the reflected light, the output interference intensity can be used to characterize the minute displacement induced by an external physical quantity [19]. The basic interference structure of the EFPI sensor is shown in Figure 1.

According to multi-beam interference theory, the intensity of the optical signal returned through the fiber can be expressed as [20]

(1) $$I(\lambda ,L)=I_{0}\cdot \frac{R_{1}+R_{2}-2\sqrt{R_{1}R_{2}}\cos\left(\frac{4\pi nL}{\lambda }\right)}{1+R_{1}R_{2}-2\sqrt{R_{1}R_{2}}\cos\left(\frac{4\pi nL}{\lambda }\right)}$$

where I_0_ is the intensity of the incident light; R_1_ and R_2_ are the reflectivities of the fiber end face and the coupled diaphragm, respectively; L is the Fabry–Perot cavity length; λ is the wavelength of the incident light; and *n* is the refractive index of the medium inside the cavity. When the medium is air, *n* = 1. When the Fabry–Perot cavity length varies periodically, the interference phase changes accordingly, resulting in a periodic variation in the reflected optical intensity. Therefore, the cavity-length modulation signal can be demodulated by detecting the variation in the reflected optical intensity.

**Figure 2:**
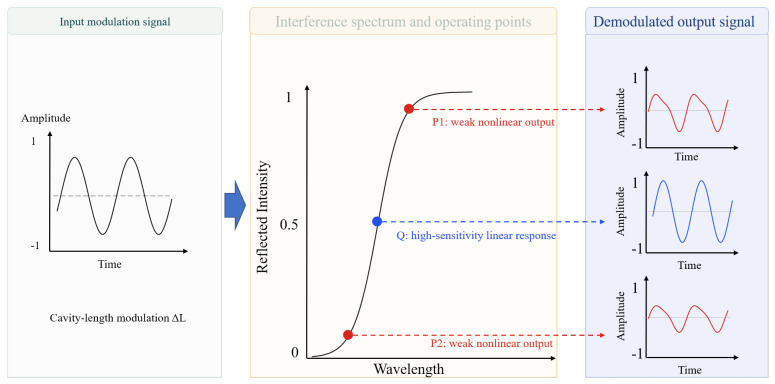
Comparison of intensity-demodulated output signals at different static operating points.

As shown in Equation (1), the reflected output intensity of the EFPI sensor is closely related to the Fabry–Perot cavity length, the incident wavelength, and the reflectivities of the two end faces. When the incident wavelength remains constant, the cavity-length variation induced by an external disturbance changes the phase difference between two or multiple reflected beams, thereby periodically modulating the output optical intensity. Therefore, when a narrowband optical signal is incident on the linear sideband region of the interference spectrum, the cavity-length variation can be converted into an optical-intensity variation, enabling sensing detection based on intensity demodulation. During intensity demodulation, the amplitude of the output signal depends not only on the cavity-length variation but also on the modulation depth of the interference spectrum itself. To illustrate the influence of static operating point selection [20] on the intensity-demodulated output signal, this study compares the output signals corresponding to different operating points under the same cavity-length perturbation. The results are shown in Figure 2.

As shown in Figure 2, under the same cavity-length perturbation, different static operating points correspond to different local slopes of the interference spectrum. This leads to significant differences in the amplitude and linearity of the intensity-demodulated output signals. When the operating point is located in the linear sideband region with a large spectral slope, the cavity-length variation can be more effectively converted into an optical-intensity variation, resulting in a larger output amplitude and better linearity. When the operating point is near a spectral peak or valley, the local slope decreases, the output amplitude is reduced, and nonlinear distortion is more likely to be introduced. Therefore, during intensity demodulation, the center wavelength of the incident narrowband light must be stabilized near the optimal static operating point.

**Figure 3:**
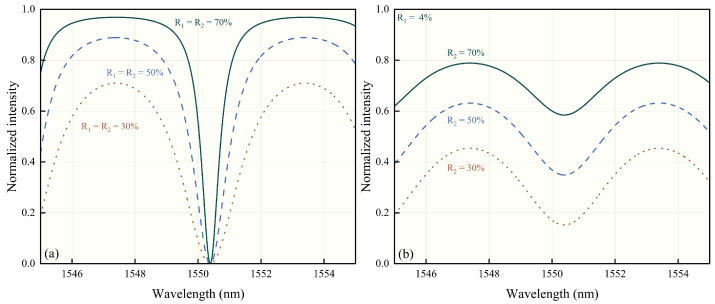
Interference spectra under different end-face reflectivity conditions. (**a**) Symmetric reflectivity condition with R_1_ = R_2_ = 70%, R_1_ = R_2_ = 50%, and R_1_ = R_2_ = 30%. (**b**) Asymmetric reflectivity condition with R_1_ = 4% and R_2_ = 70%, 50%, and 30%, respectively.

In addition to the static operating point, the modulation depth of the interference spectrum also affects the output characteristics of intensity demodulation. The finesse of the interference spectrum is mainly associated with the Fabry–Perot cavity length, whereas the modulation depth is closely related to the reflectivities of the two Fabry–Perot cavity end faces. To obtain a desirable interference-spectrum modulation depth while maintaining an appropriate linear detection range, the interference spectra under symmetric and asymmetric end-face reflectivity conditions were calculated according to Equation (1). The results are shown in Figure 3.

To further clarify the influence of end-face reflectivity on the interference-spectrum modulation depth, the interference spectra were calculated using Equation (1) under both symmetric and asymmetric reflectivity conditions. In the symmetric condition shown in Figure 3a, the two Fabry–Perot cavity end-face reflectivities were set as R_1_ = R_2_ = 70%, R_1_ = R_2_ = 50%, and R_1_ = R_2_ = 30%, respectively. In the asymmetric condition shown in Figure 3b, the reflectivity of the fiber end face was fixed at R_1_ = 4%, corresponding to the typical Fresnel reflection of an uncoated silica–air interface, while the reflectivity of the coated quartz diaphragm was set as R_2_ = 70%, R_2_ = 50%, and R_2_ = 30%, respectively. These parameter settings were used to compare the influence of reflectivity matching and reflectivity mismatch on the modulation depth of the EFPI reflection spectrum.

The calculation results show that the modulation depth of the asymmetric interference spectrum is significantly lower than that of the symmetric interference spectrum. Under the symmetric reflectivity condition, a higher reflectivity at both Fabry–Perot cavity end faces results in a larger modulation depth. However, although increasing the modulation depth can greatly improve the detection sensitivity of the sensor, it also reduces the linear detection range. According to the calculated results, when the reflectivities of both end faces are 50%, the modulation depth of the interference spectrum decreases by 15%, whereas the detection range increases by approximately 80%. Therefore, to ensure sufficient detection sensitivity while obtaining a larger measurement range, the reflectivities of both Fabry–Perot cavity end faces are selected as 50%.

### 2.2. Structural Design of the Current Transformer Based on Piezoelectric Ceramic Materials

Piezoelectric ceramic materials can generate controllable displacement responses corresponding to the driving voltage within a certain operating voltage range. Their material parameters directly affect the driving efficiency, frequency response, and long-term stability of fiber-optic current transformers. The piezoelectric ceramic material used in this study is a lead zirconate titanate piezoelectric ceramic provided by CoreMorrow Technology Co., Ltd. (Harbin, China) Its mechanical quality factor is 70, its piezoelectric constants are d_31_ = −290 pC/N and d_33_ = 635 pC/N, its Curie temperature is 150 °C, and its density is 8000 kg/m^3^. Its overall performance is generally consistent with that of PZT-5H.

To further clarify the signal conversion relationship of the proposed EFPI-PZT fiber-optic current transformer, the current-to-output response can be described by a small-signal approximation. When the measured alternating current passes through the busbar, the time-varying magnetic flux in the toroidal magnetic core induces a voltage in the pickup winding according to Faraday’s law:

(2) $$V_{\mathrm{ind}}=-N\frac{d\Phi }{dt}$$

where V_ind_ is the induced voltage applied to the PZT actuator, N is the number of turns of the induction winding, and Φ is the magnetic flux in the toroidal core. For a sinusoidal current with a fixed frequency and a fixed magnetic-core structure, the amplitude of Vind is approximately proportional to both the measured current amplitude and the number of winding turns. The axial displacement of the PZT actuator can then be approximated as

(3) $$\Delta L=k_{p}V_{\mathrm{ind}}$$

where k_p_ is the voltage–displacement coefficient of the PZT actuator. When the incident narrowband light operates on the linear sideband of the EFPI interference spectrum, the optical-intensity variation can be linearized as

(4) $$\Delta I_{\mathrm{opt}}\approx \left(\frac{\partial I}{\partial L}\right)\Delta L$$

After photodetection, the output voltage can be expressed as

(5) $$\Delta V_{\mathrm{out}}\approx G_{\mathrm{pd}}\left(\frac{\partial I}{\partial L}\right)k_{p}V_{\mathrm{ind}}$$

where G_pd_ represents the photoelectric conversion gain of the photodetector and subsequent acquisition circuit. Therefore, within the small-signal linear operating region, the output voltage is approximately proportional to the measured current. This relationship also indicates that increasing the number of winding turns N can enhance the induced voltage and improve low-current sensitivity. However, a larger N also increases the driving voltage applied to the PZT under the same measured current, which may cause a larger EFPI cavity-length modulation and make the sensor enter the nonlinear sideband region earlier. As a result, the low-current resolution and the upper limit of the linear measurement range are coupled through the selection of N.

Lead zirconate titanate is a typical ferroelectric functional material, and its piezoelectric properties are closely related to the orientation state of the electric domains inside the material. Before polarization treatment, the electric domains inside the material are randomly oriented, and the polarization effects of different microregions cancel each other out. Therefore, the material does not exhibit an obvious macroscopic piezoelectric response. After high-voltage polarization treatment, the electric domains become directionally aligned under the applied strong electric field, forming a relatively stable remanent polarization direction inside the material. When the direction of the applied electric field is consistent with the polarization direction, the material elongates along the corresponding direction. When the direction of the applied electric field is opposite to the polarization direction, the material tends to contract. In this way, mechanical deformation under an electric field is achieved.

**Figure 4:**
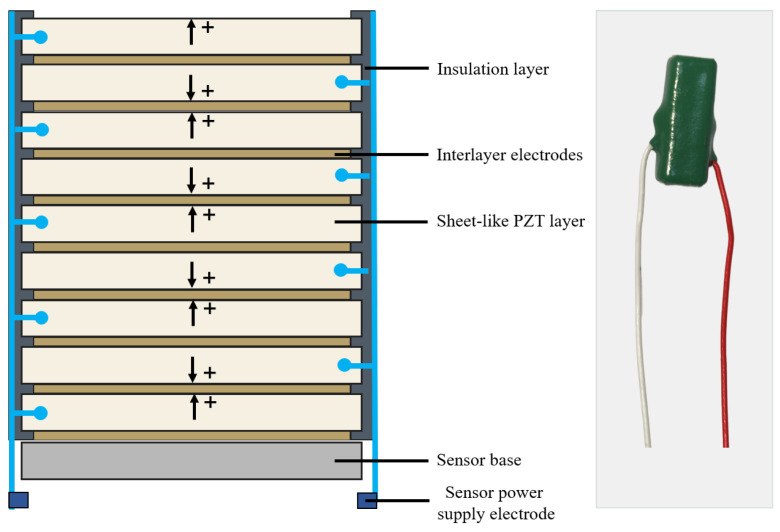
Schematic structure of the stacked PZT actuator. (**Left**) Schematic structure (**Right**) Photograph.

For the PZT actuator, its structural dimensions directly affect the required driving voltage and mechanical performance. Bulk PZT usually has relatively high mechanical strength and load-bearing capacity. However, because of its large thickness, a high voltage must be applied to obtain a sufficient electric-field strength and drive the material to produce effective displacement. In contrast, sheet-type PZT has a smaller thickness and can form a larger internal electric field under a lower voltage. Therefore, it is more suitable for low-voltage microdisplacement actuation. In the fiber-optic current transformer designed in this study, the PZT mainly functions as an electromechanical conversion and microdisplacement actuation unit, and it is not required to withstand large external loads or output large thrust. Therefore, a stacked PZT actuator is selected as the voltage–displacement conversion device. This structure features low-voltage driving and an obvious displacement response, making it suitable for microdisplacement modulation in the proposed sensing structure under the present laboratory conditions. Its long-term operating stability still requires further evaluation through repeated current cycling and packaged-sensor stability tests. The structure of the adopted PZT actuator is shown in Figure 4.

**Figure 5:**
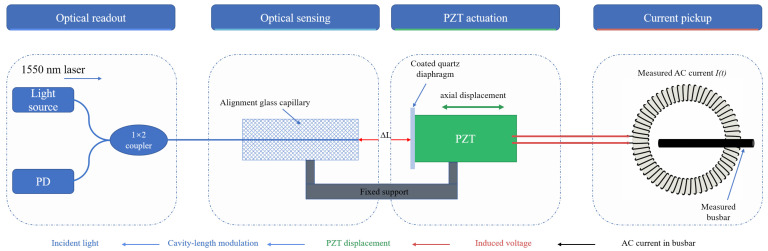
Schematic structure of the EFPI-PZT fiber-optic current transformer.

When a voltage signal is applied to the PZT actuator, the PZT generates a corresponding axial displacement. Under an AC voltage, the PZT undergoes periodic expansion and contraction around its initial equilibrium position, thereby providing microdisplacement actuation for Fabry–Perot cavity-length modulation. As shown in Figure 5, this study uses a toroidal magnetic core combined with an induction winding to convert the measured current into a driving voltage. After the measured busbar passes through the central hole of the toroidal magnetic core, the alternating current produces a time-varying magnetic flux in the magnetic core and induces an AC voltage in the outer induction winding. This induced voltage is applied to the PZT actuator through connecting wires, causing the PZT to drive the coated quartz diaphragm at its end to generate periodic displacement. The fiber pigtail is fixed in an alignment glass capillary, and the support structure ensures that the fiber end face remains parallel to the coated quartz diaphragm, thereby forming an air Fabry–Perot cavity. When the PZT expands and contracts under the induced voltage, the cavity length between the fiber end face and the coated quartz diaphragm varies periodically, enabling conversion of the measured current signal into an optical interference intensity signal.

As shown in Figure 5, the fiber-optic current transformer designed in this study uses the toroidal magnetic core and induction winding as the current pickup unit, the PZT actuator as the core component for converting the current signal into mechanical displacement, and the air Fabry–Perot cavity formed by the fiber end face and the coated quartz diaphragm as the optical sensing unit. When an alternating current flows through the measured busbar, the magnetic flux in the toroidal magnetic core varies periodically with the current and generates an AC induced voltage in the induction winding. This voltage directly acts on the PZT actuator, causing it to produce periodic axial expansion and contraction and to drive the coated quartz diaphragm at its end to move synchronously. Because the coated quartz diaphragm and the fiber end face together form the EFPI sensing cavity, the diaphragm displacement induces periodic variations in the Fabry–Perot cavity length, which in turn modulates the reflected interference intensity. By demodulating the optical signal, a voltage output corresponding to the measured current can be obtained.

## 3. Preparation and Testing of the Fiber-Optic Current Transformer

### 3.1. Static Operating Point Control Method Based on DC-Component Feedback

For FP cavity sensors with multi-beam interference characteristics, intensity demodulation based on the spectral sideband is a more direct approach. However, this demodulation method is highly susceptible to temperature interference. When the ambient temperature changes, the Fabry–Perot cavity length of the EFPI sensor varies due to thermal expansion or structural thermal drift, causing an overall shift in the interference spectrum. According to the analysis in Figure 2, the center wavelength of the incident narrowband light, which was originally matched to the optimal static operating point Q, deviates from the position with the maximum spectral slope. This reduces the sensitivity and linearity of the output optical-intensity response and consequently affects the stability of the current detection result. When the center wavelength of the incident light remains unchanged, temperature-induced drift of the interference spectrum changes not only the optical-intensity modulation amplitude under AC excitation but also the average reflected optical intensity at the operating point. Therefore, the DC component in the photodetector output signal can reflect the deviation in the current operating point from the optimal static operating point Q. Based on this relationship, the DC component of the output signal can be used as a closed-loop feedback variable to determine whether the center wavelength of the incident narrowband light remains within the optimal operating region on the interference-spectrum sideband. Therefore, to ensure that the sensor maintains the optimal demodulation state under different temperature conditions, the center wavelength of the incident narrowband optical signal must be dynamically adjusted so that it continuously follows the position of the optimal static operating point in the interference spectrum.

To achieve this objective, a broadband light source combined with a fiber Fabry–Perot tunable filter is used in this study to generate a narrowband optical signal with an adjustable center wavelength. The broadband optical signal first enters the fiber Fabry–Perot tunable filter and is filtered to produce a demodulation light signal with a narrow spectral width. When different control voltages are applied to the tunable filter, the piezoelectric actuator inside the filter produces corresponding expansion or contraction, changing the internal FP cavity length and thereby tuning the center wavelength of the transmitted narrowband optical signal. In this way, the system can continuously adjust the center wavelength of the incident demodulation light by controlling the driving voltage of the filter.

**Figure 6:**
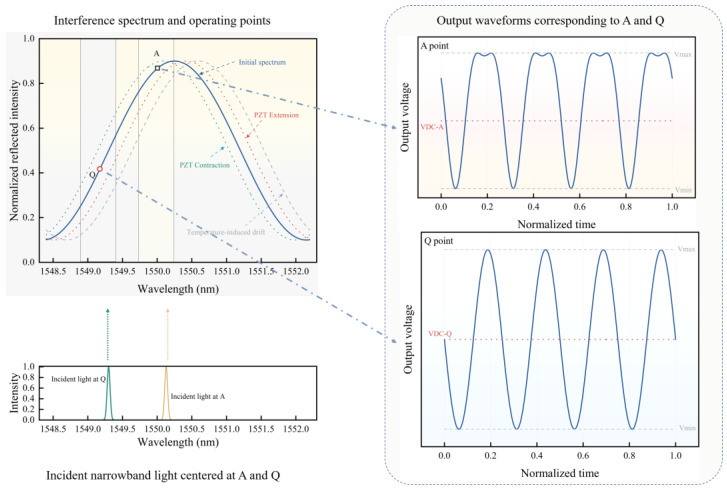
Principle of static operating point Q control based on the DC component.

During closed-loop control, the narrowband optical signal is reflected by the sensor and then converted into a voltage signal by the photodetector. When the center wavelength of the incident light coincides with the optimal static operating point Q of the sensor, and the sensor is not subjected to AC current excitation, the photodetector outputs a stable DC voltage. The amplitude of this voltage can be used as the reference baseline corresponding to the optimal operating point. When the sensor detects an AC current signal, the PZT periodically modulates the Fabry–Perot cavity length, and the photodetector output becomes an AC voltage signal superimposed on a DC bias. For this type of signal, the DC component can be approximately represented by the average of the maximum and minimum output voltages, namely ((V_max_ + V_min_)/2). Thus, the DC baseline and the AC response can be separated during signal processing. The principle is shown in Figure 6.

**Figure 7:**
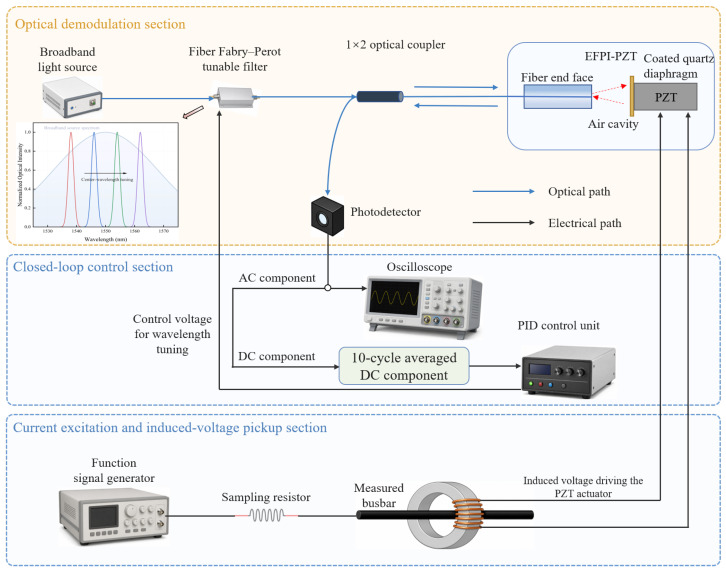
Schematic diagram of the detection system.

Because ambient temperature variation is a slow process relative to the power-frequency current signal, the voltage data from 10 consecutive cycles of the sensor output signal are averaged to improve the stability of DC-component extraction. This average value is then used as the feedback control variable and input to the PID control unit, enabling real-time adjustment of the fiber Fabry–Perot tunable filter. Using this method, the influence of operating-point drift can be reduced under relatively stable optical-link conditions. Nevertheless, because the DC component may also be affected by optical power fluctuation and link loss variation, the proposed method should be combined with reference-channel monitoring or power-normalization strategies in practical engineering applications. The structure of the sensing, detection, and demodulation system is shown in Figure 7. In the experimental system, the broadband light source is an ASE-C LIGHT SOURCE with an output wavelength range of 600–1700 nm and an output optical power of 100 mW. The fiber Fabry–Perot tunable filter is an FFP-TF2 Tunable Filter manufactured by Micron Optics. The photodetector is a PDA10CS-EC manufactured by Thorlabs, and the standard function signal generator is a UTG932 manufactured by UNI-T.

As shown in Figure 7, the optical signal emitted by the broadband light source is first filtered by the fiber Fabry–Perot tunable filter to form a narrowband demodulation light with an adjustable center wavelength. It is then coupled into the EFPI-PZT sensing probe. The interference optical signal reflected from the sensing probe is converted into a voltage signal by the photodetector. The AC component is used to characterize the Fabry–Perot cavity-length variation caused by the measured current, whereas the DC component is used to determine the matching state between the current center wavelength of the incident light and the optimal static operating point Q. When ambient temperature variation causes drift of the interference spectrum, the DC component of the output signal shifts accordingly. The control system adjusts the driving voltage of the fiber Fabry–Perot tunable filter according to this deviation, so that the center wavelength of the narrowband demodulation light tracks the optimal operating point again. Through this closed-loop feedback process, the system can maintain a relatively stable intensity-demodulation state under temperature-varying conditions, thereby reducing the influence of operating point drift on the current detection results.

It should be noted that the proposed DC-component feedback method is based on the assumption that the optical source power and the fiber-link loss remain relatively stable during the feedback interval. Under this condition, the variation in the DC component can mainly reflect the relative deviation between the incident narrowband wavelength and the EFPI interference spectrum. However, in a practical intensity-demodulated system, the DC component may also be affected by common-mode optical power variations, including source–power fluctuation, source aging, connector instability, and bending-induced fiber loss. Therefore, the present DC-component feedback method cannot independently distinguish optical power fluctuation from temperature-induced spectral drift if only a single sensing optical path is used. In this study, the method is regarded as a preliminary operating-point stabilization strategy under controlled optical-link conditions rather than a complete compensation scheme for all intensity fluctuation sources. For future engineering applications, an additional reference optical channel, optical power normalization, or differential detection scheme should be introduced to separate common-mode optical power variations from EFPI spectral drift, thereby improving the robustness of the operating-point control.

### 3.2. Sensor Preparation and Testing

**Figure 8:**
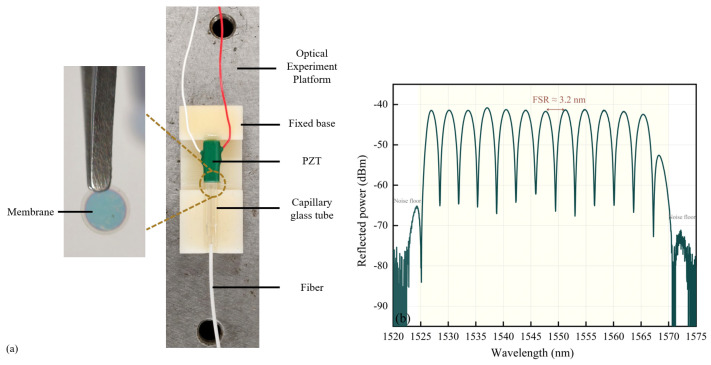
Fabricated sensor and its reflected interference spectrum: (**a**) photograph of the sensor; (**b**) reflected interference spectrum.

To verify the feasibility of the proposed EFPI-PZT fiber-optic current transformer, the sensing probe was first fabricated, and its reflected interference spectrum and amplitude–frequency response characteristics were tested. The fixed support structure of the sensor was fabricated by 3D printing to realize the relative positioning among the PZT actuator, coated quartz diaphragm, alignment glass capillary, and fiber pigtail. During fabrication, the coated quartz diaphragm was fixed to the end face of the PZT actuator, and the fiber pigtail was inserted into the alignment glass capillary so that the fiber end face and the coated quartz diaphragm together formed an air Fabry–Perot cavity. Then, a precision three-dimensional displacement stage was used to adjust the spacing between the fiber end face and the coated quartz diaphragm. During adjustment, the reflected interference spectrum of the sensor was monitored in real time using an optical spectrum analyzer. After relatively clear periodic peaks and valleys appeared in the interference spectrum, ultraviolet-curable adhesive was used to fix the fiber pigtail and the alignment glass capillary. The fabricated sensor and its reflected interference spectrum are shown in Figure 8.

As shown in Figure 8b, the fabricated sensor presents a clear periodic reflected interference spectrum, indicating that an effective air Fabry–Perot cavity was formed between the fiber end face and the coated quartz diaphragm. To further verify the rationality of the measured interference spectrum, the initial cavity length was estimated from the free spectral range (FSR) of the reflected spectrum. For an air Fabry–Perot cavity, the relationship between the cavity length and FSR can be expressed as

(6) $$L_{0}={\lambda_{0}}^{2}/(2n\cdot FSR)$$

where L_0_ is the initial cavity length, λ_0_ is the central wavelength used for the estimation, *n* is the refractive index of the cavity medium, and FSR is the wavelength spacing between adjacent interference peaks. Since the cavity medium is air, *n* ≈ 1. According to the adjacent interference peaks in Figure 8b, the experimental FSR was approximately 3.2 nm near 1550 nm. Therefore, the initial cavity length was estimated to be approximately 375 μm. Substituting this estimated cavity length back into the FSR expression gives a theoretical FSR of approximately 3.2 nm, which agrees with the experimentally observed spectral spacing in Figure 8b. This comparison confirms that the observed reflected spectrum is consistent with the expected EFPI interference behavior and provides a quantitative basis for subsequent intensity demodulation.

**Figure 9:**
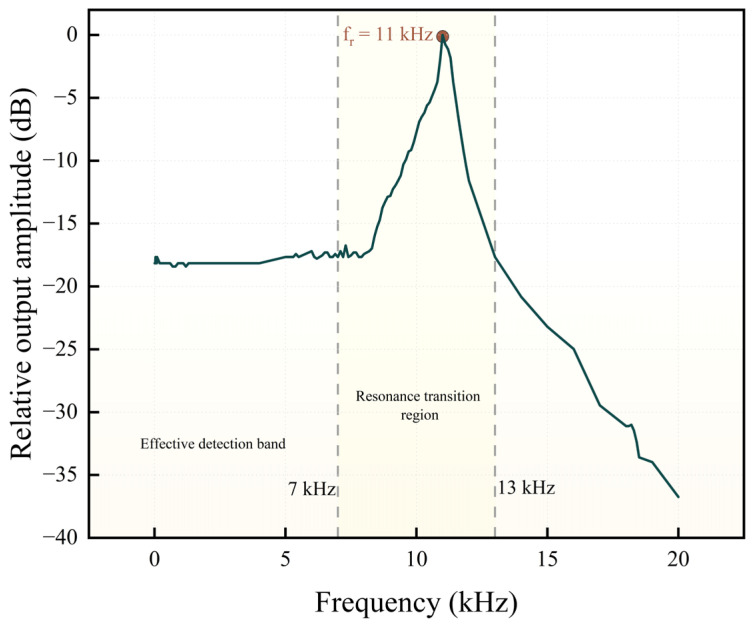
Amplitude–frequency response curve of the sensor.

Before conducting the current detection experiment, the frequency response characteristics of the sensing probe were further tested to determine its effective operating bandwidth. This experiment was mainly used to characterize the amplitude–frequency response of the EFPI-PZT sensing probe under direct voltage driving; therefore, the toroidal magnetic induction structure was not introduced. In the experiment, a digital signal generator was used to directly apply an AC voltage to the PZT actuator. The driving signal frequency was gradually increased, and the peak-to-peak output voltage of the photodetector at different frequencies was recorded. The amplitude–frequency response curve of the sensor was then obtained, as shown in Figure 9.

As shown in Figure 9, in the low-frequency range, the peak-to-peak value of the sensor output signal changes only slightly with the driving frequency, indicating that the sensing probe has a relatively stable response within this frequency range. When the driving frequency increases to approximately 7 kHz, the output peak-to-peak value begins to increase. When the driving frequency approaches 11 kHz, the sensor exhibits an obvious resonance, and the output amplitude increases significantly. As the frequency further increases beyond 13 kHz, the output amplitude gradually decreases, indicating that the sensor gradually moves away from the resonant response region. To avoid the influence of the resonant region on the current detection signal, the effective detection bandwidth of the sensor is determined to be 0–7 kHz. Since the power-frequency current signal is 50 Hz and lies within the stable response range of the sensor, the fabricated sensor can meet the requirements of subsequent power-frequency low-current detection experiments.

**Figure 10:**
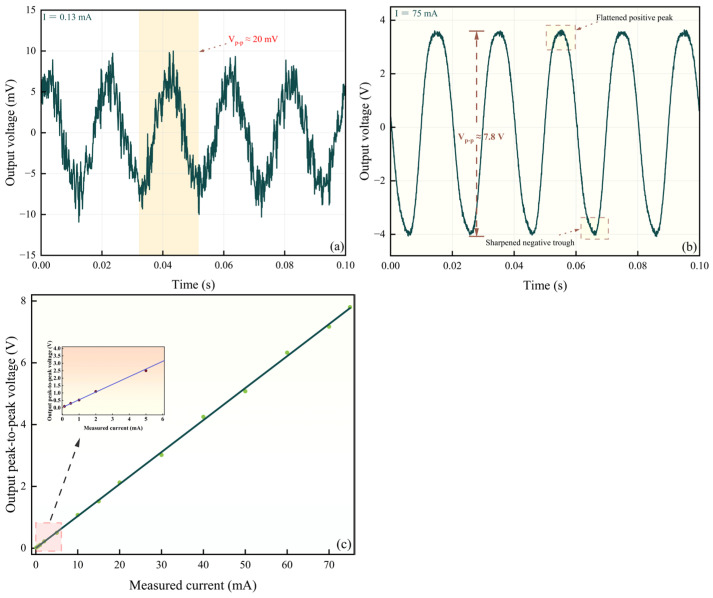
Output characteristics of the sensor under different measured currents: (**a**) output waveform under a low current of 0.13 mA; (**b**) output waveform under a high current of 75 mA; (**c**) relationship between the output peak-to-peak value and the measured current.

According to the experimental system shown in Figure 7, a fiber-optic current transformer experimental platform was established. The system mainly consists of a function signal generator, a measured busbar, a sampling resistor, a toroidal magnetic induction structure, an EFPI-PZT sensing probe, a broadband light source, a fiber Fabry–Perot tunable filter, a photodetector, and an oscilloscope. The toroidal magnetic induction structure has an inner diameter of 5.1 cm, an outer diameter of 8.3 cm, and a height of 2.6 cm. The winding has 220 turns, and the coil resistance is 0.88 Ω. During testing, the function signal generator output a power-frequency sinusoidal voltage to the test circuit, forming a corresponding power-frequency sinusoidal current in the series sampling resistor and the measured busbar. After the measured busbar passed through the central hole of the toroidal magnetic core, an induced voltage corresponding to the measured current was generated in the winding of the magnetic structure. This induced voltage was directly applied to the PZT actuator, causing the PZT to generate periodic axial expansion and contraction. The coated quartz diaphragm at the end of the PZT was then driven to move synchronously, resulting in periodic variation in the EFPI cavity length. Subsequently, the cavity-length modulation information was converted into a voltage signal through photodetection and intensity demodulation and was collected by the oscilloscope. Because the measured busbar itself has a small resistance, it is difficult to obtain a stable small current by directly applying a voltage from the function signal generator. Therefore, a 250 Ω sampling resistor was connected in series in the test circuit. During the experiment, the voltage across the sampling resistor was synchronously measured by the oscilloscope, and the actual measured current was calculated according to Ohm’s law. By gradually adjusting the output voltage of the function signal generator, the sensor output voltage waveforms under different current excitations were obtained. The test results are shown in Figure 10.

As shown in Figure 10a, when the measured current was 0.13 mA, the peak-to-peak value of the sensor output signal was approximately 20 mV. The observed system noise floor under the present experimental condition was approximately 5 mV. Therefore, the corresponding peak-to-peak signal-to-noise ratio can be approximately estimated as 20 mV/5 mV = 4. This result indicates that the output waveform at 0.13 mA was still distinguishable from the background noise under the present setup. However, because the strict limit of detection should be determined from repeated noise measurements, statistical standard deviation, and a defined confidence criterion, 0.13 mA is described in the revised manuscript as the minimum distinguishable current under the present experimental conditions rather than as an absolute detection limit.

Using the low-current response at 0.13 mA as a rough estimate, the local peak-to-peak sensitivity is approximately 20 mV/0.13 mA = 153.8 mV/mA. Based on the observed noise floor of approximately 5 mV, the corresponding noise-equivalent current can be estimated to be approximately 0.033 mA under the present measurement condition. This value is only an approximate noise-based estimate and should not be regarded as a rigorously characterized resolution limit without repeated statistical measurements.

When the measured current increased to 75 mA, the peak-to-peak output voltage reached approximately 7.8 V, and waveform distortion appeared, including flattening in the positive half-cycle and a sharp variation in the negative half-cycle. This phenomenon indicates that the EFPI-PZT sensor was approaching the boundary of the linear intensity-demodulation region. According to the current-to-output relationship described above, increasing the winding turns N can improve the induced voltage and low-current response, but it also increases the PZT displacement for the same measured current. Excessive cavity-length modulation may move the operating point away from the linear sideband region of the interference spectrum, leading to premature nonlinear distortion and a reduced linear measurement range. Therefore, the selection of N should be regarded as a trade-off between low-current resolution and linear dynamic range. In this study, N = 220 was used to obtain a clear low-current response under laboratory conditions. Further optimization of N, magnetic-core parameters, and EFPI operating point should be carried out in future work to quantitatively balance sensitivity, resolution, and measurement range.

### 3.3. Research Outlook

The EFPI-PZT fiber-optic current transformer designed in this study provides a certain degree of parameter tunability. The detection sensitivity and effective measurement range can be adjusted by optimizing the Fabry–Perot cavity length, end-face reflectivity, toroidal magnetic-core dimensions, and number of induction-winding turns. However, because the proposed sensor relies on the inverse piezoelectric deformation of the PZT actuator to modulate the EFPI cavity length, the long-term mechanical stability of the PZT-coupled structure is an important factor for practical application. During repeated current detection, PZT hysteresis, creep, fatigue, bonding-layer stability, and diaphragm–PZT coupling efficiency may affect the voltage–displacement relationship and further influence the repeatability of the EFPI cavity-length modulation. In the present work, the feasibility of the EFPI-PZT current-sensing mechanism was verified under controlled laboratory conditions, but long-term cyclic loading, hysteresis characterization, and fatigue-related response degradation were not systematically evaluated. Therefore, the present results should be regarded as a preliminary validation of the sensing principle rather than a complete reliability assessment.

In addition, practical environmental disturbances may also influence the performance of the proposed sensor. Temperature variation may cause thermal expansion of the EFPI cavity structure, change the voltage–displacement coefficient of the PZT actuator, and affect the bonding state between the PZT and the coated quartz diaphragm. External mechanical vibration may introduce additional cavity-length perturbations, which can be superimposed on the current-induced EFPI modulation signal. Although the optical fiber link has strong immunity to electromagnetic interference, the toroidal magnetic core, induction winding, photodetector, and acquisition circuit may still be affected by strong electromagnetic environments in practical power systems. Connector stability, fiber bending loss, package stiffness, and adhesive aging may also change the optical link loss or displacement-transfer efficiency, thereby affecting the output baseline and measurement repeatability. Therefore, future work should further investigate repeated current cycling, long-duration operation, PZT hysteresis compensation, temperature-dependent response drift, vibration-induced noise, electromagnetic shielding of peripheral circuits, fiber-link power fluctuation, and packaged-sensor stability. These studies are necessary before the proposed sensing structure can be applied to long-term current monitoring in practical power equipment.

## 4. Conclusions

This study proposed a fiber-optic current transformer based on the coupling of an EFPI and a PZT. A toroidal magnetic core and an induction winding were used to convert the measured alternating current into an induced voltage, which directly drove the PZT to generate axial displacement. This periodically modulated the Fabry–Perot cavity length, thereby converting the current signal into an optical interference intensity signal. By analyzing the EFPI interference characteristics, static operating point, and end-face reflectivity, the sensing-structure parameters suitable for intensity demodulation were determined, and the EFPI-PZT sensing probe was fabricated.

The experimental results show that the fabricated sensor can generate a stable reflected interference spectrum and exhibits a relatively flat frequency response within the range of 0–7 kHz, indicating its potential for power-frequency current detection under the present laboratory conditions. The current detection results show that when the measured current is 0.13 mA, the sensor can still output a distinguishable sinusoidal signal. When the current increases to 75 mA, nonlinear distortion begins to appear in the output signal, indicating that the sensor is approaching the boundary of its linear detection range. Within the linear operating region, the output peak-to-peak value shows good linear variation with the increase in the measured current. Although the proposed EFPI-PZT structure shows a clear low-current response under the present laboratory conditions, its long-term repeatability, PZT hysteresis, fatigue behavior, and packaged stability still require further systematic evaluation before practical engineering application.

In addition, this study proposed an operating point control method based on DC-component feedback. By adjusting the driving voltage of the fiber Fabry–Perot tunable filter, the center wavelength of the incident narrowband light can track the optimal operating point of the interference spectrum. This provides a possible operating-point stabilization strategy under relatively stable optical-link conditions. However, because the DC component may also be affected by optical power fluctuation and fiber-link loss variation, systematic temperature-varying experiments and power-normalization strategies are still required for future engineering applications. Overall, the proposed EFPI-PZT fiber-optic current transformer has the advantages of a relatively simple structure, clear low-current response, and adjustable structural parameters, providing a reference for the miniaturized design of new fiber-optic current sensors. However, these results should still be regarded as a laboratory-stage validation of the sensing principle.

## Data Availability

The original contributions presented in this study are included in the article. Further inquiries can be directed to the corresponding author.

## References

[1] Tang M., Li R., Zhang R., Yang S. (2024). Research on New Electric Power System Risk Assessment Based on Cloud Model. Sustainability.

[2] Swain A., Abdellatif E., Mousa A., Pong P.W.T. (2022). Sensor Technologies for Transmission and Distribution Systems: A Review of the Latest Developments. Energies.

[3] Zhu S., Yu T., Ying Y., Zhao Z., Zhao D. (2019). New optical differential protection algorithm applied in bus protection. J. Eng..

[4] Wu J., Zhang X. (2020). Recent Progress of All Fiber Optic Current Transformers. Proceedings of the 2020 7th International Forum on Electrical Engineering and Automation (IFEEA).

[5] Zhang Z., Zhang X., Li B., Qin S., Ding L. (2024). Temperature characterization of fiber optic current sensor influenced by polarization-maintaining transmission fiber. Sens. Actuators A Phys..

[6] Zhu X., Zhao X., Liu X., Ren Z. (2024). Temperature stability analysis of the all-fiber current sensor with a loop structure. Laser Phys..

[7] Yu A., Huang Y., Li S., Wang Z., Xia L. (2023). All fiber optic current sensor based on phase-shift fiber loop ringdown structure. Opt. Lett..

[8] Zhang S., Liu Q., Chang Y., He Z. (2024). Resonant Fiber Optic Current Sensor Based on Broadband Light Source. J. Light. Technol..

[9] Chen H., Guan C., Lv H., Guo C., Chai S. (2023). Improved Optical Path Structure for Symmetric Demodulation Method in EFPI Fiber Optic Acoustic Sensors Using Wavelength Division Multiplexing. Sensors.

[10] Mir B., Niewczas P., Fusiek G. (2023). Design and Implementation of a Passive Autoranging Circuit for Hybrid FBG-PZT Photonic Current Transducer. Sensors.

[11] Fusiek G., Mir B., Niewczas P. (2024). The Performance of a Passive Autoranging Method for a Photonic Current Transducer. Sensors.

[12] Sun S., Ma F., He Y., Niu B., Wang C., Dai L., Zhao Z. (2025). An Optimized PZT-FBG Voltage/Temperature Sensor. Micromachines.

[13] Dante A., Lopez J.D., Carvalho C.C., Allil R.C.S.B., Werneck M.M. (2019). A Compact FBG-Based Magnetostrictive Optical Current Sensor With Reduced Mass of Terfenol-D. IEEE Photonics Technol. Lett..

[14] Jiao F., Lei Y., Peng G., Dong F., Yang Q., Liao W. (2023). Grating Spectrum Design and Optimization of GMM-FBG Current Sensor. Energies.

[15] Zhuang Y., Xie T., Cai X., Wang Y., Zhou J., Liu S., Liu W., Chen S., Wang H., Fu H. (2025). Fiber-optic current sensing system by using optical carrier microwave interferometry technology and virtual Vernier effect. Opt. Laser Technol..

[16] Yang Q., He Y., Sun S., Luo M., Han R. (2017). An optical fiber Bragg grating and piezoelectric ceramic voltage sensor. Rev. Sci. Instrum..

[17] He Y., Yang Q., Sun S., Luo M., Liu R., Peng G.-D. (2020). A multi-point voltage sensing system based on PZT and FBG. Int. J. Electr. Power Energy Syst..

[18] Zhang S., Peng M., Dong J., Li S., Ren Q. (2023). Three-wavelength phase demodulation technique for extrinsic Fabry–Perot interferometric sensors. Opt. Fiber Technol..

[19] Li Z., Cui J., Chen H., Lu H., Zhou F., Rocha P.R.F., Yang C. (2025). Research Progress of All-Fiber Optic Current Transformers in Novel Power Systems: A Review. Microw. Opt. Technol. Lett..

[20] Jia J., Jiang Y., Zhang L., Gao H., Wang S., Jiang L. (2018). Dual-Wavelength DC Compensation Technique for the Demodulation of EFPI Fiber Sensors. IEEE Photonics Technol. Lett..

